# Acceptance and adoption of transoral robotic surgery in Germany

**DOI:** 10.1007/s00405-021-06623-w

**Published:** 2021-02-07

**Authors:** Magis Mandapathil, Jens E. Meyer

**Affiliations:** Department of Otorhinolaryngology, Head and Neck Surgery, Asklepios Hospital St. Georg, Lohmühlenstraße 5, 20099 Hamburg, Germany

**Keywords:** Robotic surgery, Head and neck surgery, TORS, Germany

## Abstract

**Purpose:**

Since its introduction over a decade ago, the use of robotic surgery (RS) in head and neck surgery has widely spread around the globe, with very differential adoption of this novel surgical technique in different parts of the world. In this study, we analyze the acceptance and adoption of robotic surgery in the head and neck in Germany.

**Materials and methods:**

A cross-sectional analysis using a questionnaire evaluating the acceptance and adoption of RS was performed. Questionnaires were distributed to all chairmen /-women of Otorhinolaryngology, Head and Neck Surgery Departments in Germany.

**Results:**

A total of 107 respondents completed the questionnaire (65.2%). At university hospitals, 71.4% of the respondents indicated that a robotic system was available, and 21.4% responded that robotic surgery was performed at their institution; 22.7% and 0.04%, respectively, at non-university hospitals. The overall adoption rate was 0.8%. The most common cases performed were TORS resection in the oropharynx. Main reasons for not adopting this technique were costs, lack of interest and available co-operations.

**Conclusion:**

This study provides evidence of the extent of adoption of TORS in Germany; main perceived barriers to adoption are costs with lack of cost-covering reimbursement and insufficient co-operations with other disciplines as well as hospital administration resulting in a very low adoption rate of this technique over the past decade. Results from this study may assist in decision-making processes on adopting this technique in the future.

## Introduction

Following approval of transoral robotic surgery (TORS) for resection of tumors in the oropharynx by the FDA in 2009, this technique had been adopted globally [1, 2]. Indications for its use have ever since extended to various subsites of the head and neck [3, 4] also beyond TORS, allowing, e.g. remote access surgery for performing neck dissections and thyroidectomies [5, 6]. The daVinci® surgical robot (Intuitive Surgical® Inc, Sunnyvale, CA, USA), allowing for a magnified and three-dimensional view through a dual endoscope, tremor filter, scaling of robotic arm movements and range of movement, is hereby the most commonly used robotic system (RS). But novel platforms, such as the Flex® Robotic System (Medrobotics® Cooperation, Raynham, MA, USA) particularly addressing obstacles in the head and neck, are also becoming more popular for performing TORS [7, 8]. Multiple large prospective studies have indicated safety and demonstrated favorable prognostic and functional outcome of TORS in treatment of patients with head and neck squamous cell carcinoma (HNSCC) [9, 10].

Under right circumstances, adoption of a new technology usually follows cumulative normal distribution after adoption by innovators and early users [11], with TORS being in the early stages of adoption. However, different parts of the world appear to adopt TORS into their clinical practice quite differently. Various perceptive barriers appear to be the reason hereby, including differences in health care systems, surgeons’ preferences, costs and access.

Germany had historically been well adopted to performing transoral procedures for decades due to the extended use of transoral laser microsurgery (TLM) in resection of head and neck tumors of the pharynx and larynx. Following publication of using this technique by Steiner et al. in resection of laryngeal carcinoma in 1993 [12], indications for its use have been broadened [13, 14] and ever since gained popularity not only in Germany, but worldwide. Different, is the enthusiam to adopt and perform TORS. The limited number of available systems per hospital, high acquisition and maintainance costs nearly always demands an otolaryngology department to cooperate with other surgical subspecilties in using the robot with potentially resulting in financial and logistic challanges [15, 16].

There is currently no data on the adoption of TORS in Germany. Given the established advantages and disadvantages of this technology, we aim to evaluate the use and acceptance of RS, and specifically TORS, among Otorhinolaryngology Departments in Germany in this study.

## Materials and methods

### Questionnaire

A questionnaire evaluating the use and acceptance of robotic surgery was designed by the authors and consisted of eight questions to evaluate five major categories of interest:

(1) background of the responding party [university hospital versus non-university hospital]; (2) current potential access to a robotic system; (3) characteristics of established robotic programs; (4) motivation to start a robotic program, (5) reasons for hesitations to start a robotic program; (6) inquiry of patients and referring doctors for robotic-assisted procedures.

Questionnaires were distributed by mail and responses were anonymously provided by mail as well. Although the reliability and reproducibility of the questions were not explicitly tested, the questions were based on those used in similar studies.

### Data acquisition and analysis

Questionnaires were send to all chairwomen/-men of Otolaryngology Departments throughout Germany to gather information about the use of RS within their departments. Contact information for all Departments of Otorhinolaryngology in Germany was obtained from the list provided online on the official website of the German Society of Otorhinolaryngology, Head and Neck Surgery (as of December 2019) separated into university hospitals and non-university hospitals*.* The survey was conducted anonymously, and all collected data were de-identified if necessary. For the purpose of analysis, responses were tabulated and percentages were calculated.

## Results

### Data collection

**Fig. 1:**
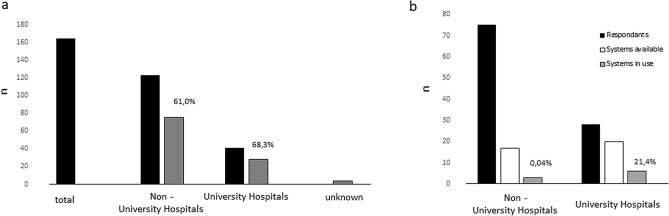
Respondents’ characteristics and robotic system availability. **a** Affiliation and percentage of responding parties is shown. **b** Available systems as well as systems in use are shown

Questionnaires were sent by mail to all chairwomen/-men of Otolaryngology, Head and Neck Surgery Departments in Germany as described in Material and Methods (*n* = 164). A total of 107 responded (response rate of 65.2%) and returned the completed questionnaire anonymously by mail. 75/123 (60.1%) responses were received from non-university hospitals and 28/41 (68.3%) from university hospitals. Four respondants did not indicate their affiliation (Fig. 1a).

### Availablity and use of a robotic system

As shown in Fig. 1b, from 28 university hospitals, which responded, 20 had a RS available at their hospital with six departments having a RS in use. Three departments had plans to use their system in the near future, and additional ten departments were potentially interested in using the RS at their hospital. Two indicated that they were not interested in using the RS present at their hospital.

Out of the 75 non-university hospitals, which responded, 17 indicated that their hospital was equiped with a RS with three departements using their RS (Fig. 1b). Two indiacted that they were interested, with plans to establish a robotic program in the near future. Seven respondants were generally interested and five responded that they were not interested in using the RS at their institution. Therefore, the adoption rate at university hospitals was 21.4% and at non-university hospitals 0.04%. The overall adoption rate was 0.8%. Noteably, at university hospitals without a RS in place (*n* = 8), seven respondants stated that they were intersted in establishing a TORS program; at non-university hospitals without a RS (*n* = 58), 28 respondants were intersted in using a robot. As per respondants, three university hospitals were in the process of purchasing a RS, and five non-university hospitals.

The main procedures performed were TORS resections of tumors in the oropharynx followed by TORS for supraglottic lesions and treatment of obstructive sleep apnea syndrome (OSAS). At two institutions, remote access surgery for thyroidectomies using the RS was performed (data not shown).

### Reasons for not adapting TORS

**Fig. 2:**
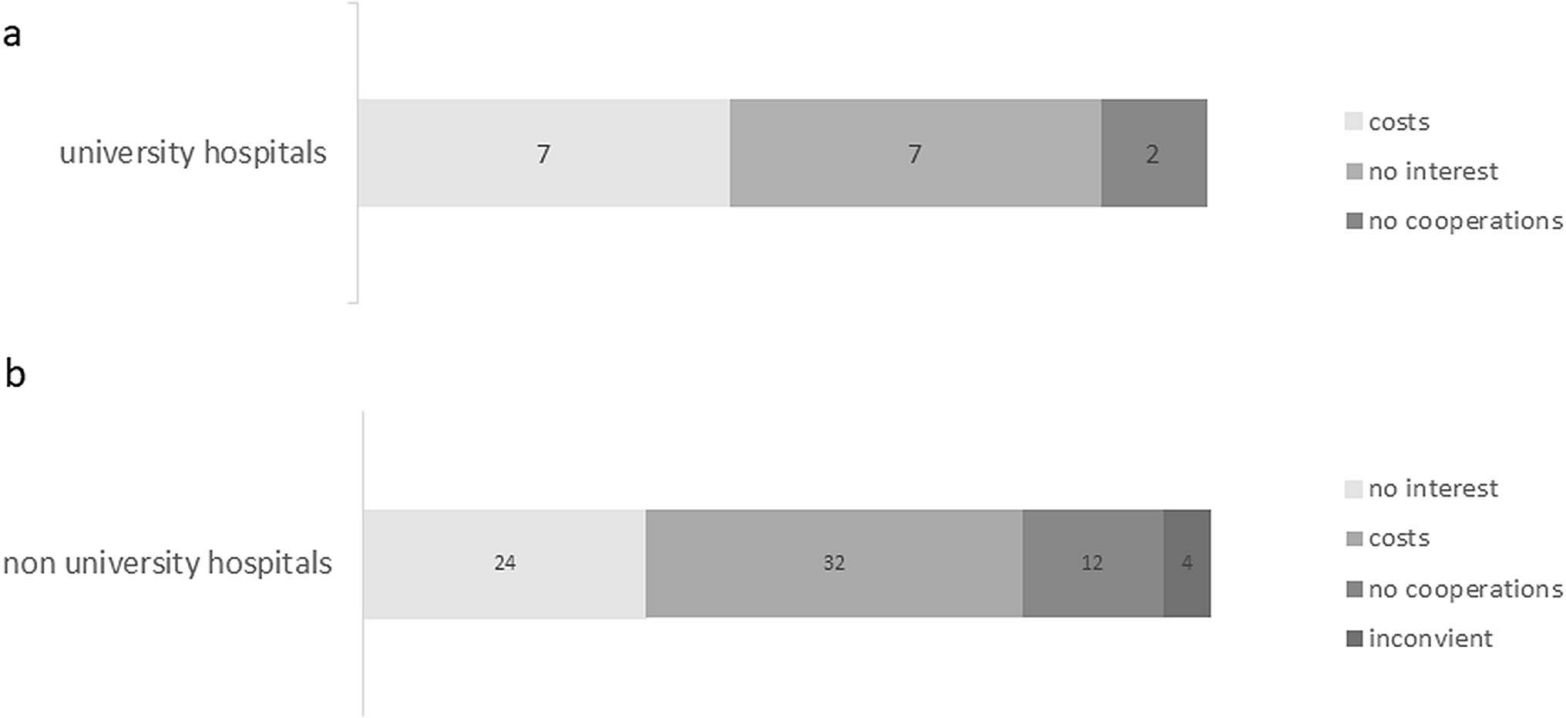
Reasons for not establishing a robotic program. Main reasons for not adopting TORS at university hospitals (**a**) and non-university hospitals (**b**) is shown. The questionnaire allowed for multiple answers by one respondent

Based on the provided answers from the respondants at university hospitals, the main barriers to implement TORS were costs, and also the same number of resondants indicated that they were not interested in implementing TORS. Lack of cooperation was further perceived as an obstacle in a smaller group of respondants (Fig. 2a). For respondants from non-university hospitals, the main concerns were costs and lack of available cooperation (Fig. 2b). In written statements provided by the respondants, many stated that this technology would not expand the scope of their surgical practice. This relates to a significant group of resondants in both cohorts to indicate their lack of interest (Fig. 2a and b). Further, respondants indicated a lack of support from hospital administration.

Among university hospitals as well as non-university hospitals, 26 respondants (25.3%) indicated that patients were actively inquiring about robotic surgery either frequently or rarely. 24 respondants (23.3%) indicated that referring doctors were inquiring about robotic surgery either frequently or rarely (Table 1). Four respondants choose not to answer.

**Table 1:** Inquire of patients and referring doctors

Inquire about robotic surgery	
Patients	Frequency
Yes	8 (7.8%)
Seldom	18 (17.5%)
No	77 (74.8%)

Referring doctors	
Yes	7 (6.8%)
Seldom	17 (16.5%)
No	79 (76.7%)

Request for robotic surgery from patients and as wells as referring doctors for university hospitals and non-university hospitals is shown

## Discussion

This work presents data characterizing the adoption of robotic surgery as well as the acceptance of this technique within Otorhinolaryngology Departments in Germany as first of its kind. With a response rate of 65.2% of all otolaryngology departments within Germany, participation in this study is satisfying. Data of this study were obtained from a voluntary-based survey with obvious limitation, such as respontant bias as well as lack of response from 57 parties. TORS for procedures in the oropharnyx was the predominant procedures performed. Estalishing a robotic program is more commonly observed in academic centers with an adoption rate of 21.4% compared to 0.04% at non-university hospitals. The overall adoption rate of 0.8% among otolaryngology departments in Germany appears relatively low in comparism with other developed countries like the United States, Australia, New Zealand and South Korea [17–21]. Currently, there are no data on adoption rates of TORS in other European countries for comparison yet. To date, two studies from the United States and one from Australia, have looked at the adoption of TORS. Chen et al. retrospectively reviewed the US National Cancer Database (US NCDB) of all adults with Oropharyngeal Squamous Cell Cancer (OPSCC) treated between 2010 and 2011 and demonstrated a 67% increase in the use of TORS at academic centers and community centers and was associated with a lower rate of positive margins compared to non-robotic surgery [20]. Cracchiolo et al. retrospectively reviewed the US NCDB from 2010 to 2013 for cases of T1, T2 and T3 OPSCC treated with surgery and described a 28% utilization of TORS predominatly for low tumour stages and at academic center [17].

Krishnan et al. demonstrated in results based on a voluntary-based online survey among otolaryngologists and head and neck surgeons in Australia and New Zealand, that 43.6% of head and neck surgeons had performed TORS, mainly for procedures in the oropharynx [lateral pharyngectomy and base of tonge resections] [18]. In an attempt to compare, Germany is hereby in an unique situation, as nearly all otolaryngology departments had established TLM programs at time of introduction of TORS.

A significant number of respondants argued that they are quite adopted and pleased with TLM for most of their transoral procedures and therefore do not feel the need for an additional tool to perform these procedures. To date, no data from multicenter randomized controlled trials comparing outcomes of TLM and TORS exist; however, there is evidence that TORS could be superior to TLM in functional outcomes in a subgroup of HNSCC patients [21]. Remote access thyroid surgery using a RS was only performed at two institution. This might be also partially be due the fact that thyroid surgery is mainly performed at General Surgery departments in Germany.

There are currently no standards in accreditation and credentialing of TORS in Germany, other than, e.g. in Australia, where the Australian Society of Otolaryngology, Head and Neck Surgery defined clear guidelines with a minimum of 20 procedures per year required to be performed by the surgeon to be accredited [18]. Results from a US study describing a novel credentialing and quality assurance process to support multicenter transoral head and neck oncology trials, showed low incidences of positive margins and grade III/IV bleeding in surgeons with ≥ 20 transoral resection for OPCC [22]. The presented survey does not allow for evaluation of each respondants/institutions case load, as it was not subject of this study. However, establishing TORS training guidelines and facilities allowing for introduction and training of interested surgeons by adopters might allow this technology to become more accessable and intersting to a broader group. Further, exposure to TORS during residency for younger surgeons, also in form of clinical rotations, surgical courses and/or using simulators, to gain experinences with robotic surgery early on in their career could help providing more practical insights into this technique and therefore create a larger community of robotic surgeons who can potentially address and tackle perceived obstacles in communication with collegues, industry and hospital adminstrations.

A major limitation for institutions performing TORS appears to be the cost of the robot and its maintainance as well as support from hospital administration, although studies suggest TORS to be a cost-effective modality in terms of lower overall treatment-related costs (ower use of adjuvant radiochemotherapy, late gastrostomy and tracheostomy) [23, 24]. However, with most of these studies being published from institutions outside Germany, these arguements, in respect to the german health care system as well as reimbursement methods, do not necessarly allow this calculation to appear entirely conclusive to Otolaryngology Departments in Germany as well hospital adminstrations. Further, there is still no established precedure code for appropriate reimbursement for the use of robotic surgery in the head and neck leaving most performed cases not economically profitable for hospitals. Performig these cases with additional costs for the patients outside insurance coverage is not a very common practice in Germany compared to other countries with approximately 85% of the overall population being insured by public health insurance. This rate might presumably be even higher in the group of patients who need treatment for head and neck malignancies. However, holistic interpretation of costs seems particularly of importance in dicussions with hospital administration as using TORS in the treatment of HNSCC patients, with higher intraoperative costs but reduced postoperative costs could result in comparable overall treatment costs in selected cases. Further, with novel RS arising, there is a possibility for cost reduction from market competition resulting in improvement in this technique.

Additionally, collaboration with other disciplines appears to be a crucial barrier to adopting this technology. Our data suggest that departments within University Hospitals appear to be able to tackle these challenges more efficiently as these departments appear to be more likely to establish a TORS program compared to non-university hospitals. This is similar to other countries, where academic centers implement TORS more frequently compared to community hospitals [17, 18], partly probably due to superior access to support from internal and external funding. Further, establishment of interdisciplinary collaborations might be an issue at some non-university hospitals, as relevant disciplines are absent. However, as resources of hospitals for funds and case load are limited, these co-operations are essential in introducing and maintaining a robotic program in Germany, not only for otolaryngology departments, but also for the other surgical disciplines involved [25].

In this study, we aim to present the current adoption and perception status for TORS in Germany. Within the limitation of our voluntary survey-based design, the results of this study show that RS among otolaryngologists in Germany is used only at a very limited number of institutions, whereas transoral approaches are nearly performed at every single institution in form of TLM. Costs, lack of effective collaborations and availablity appears to be the major barriers limiting adoption and/or expansion of TORS programs. Experiences of early adopters, exchange of skills and training and educating junior surgeons early on in their career could aid in overcoming perceived obstacles in the future. To understand trends in adoption of TORS in Germany, this study would need to be repeated in a timely manner, as the data from this study only represent a snapshot of the current moment. Experience from early, current and future adopters will shape the adoption curve and future studies will identify trends in diffusion of TORS in not only Germany, but worldwide.
